# Non-Fourier energy transmission in power-law hybrid nanofluid flow over a moving sheet

**DOI:** 10.1038/s41598-022-14720-x

**Published:** 2022-06-21

**Authors:** Awatif Alhowaity, Muhammad Bilal, Haneen Hamam, M. M. Alqarni, Kanit Mukdasai, Aatif Ali

**Affiliations:** 1 Department of Mathematics, College of Science and Arts at Alkamil, University of Jeddah, Jeddah, Saudi Arabia; 2 Department of Mathematics, City University of Science and Information Technology, Peshawar, 25000 Pakistan; 3 Mathematics Department, Umm Al-Qura University, Makkah, Saudi Arabia; 4 Department of Mathematics, College of Sciences, King Khalid University, Abha, 61413 Saudi Arabia; 5 Department of Mathematics, Faculty of Science, Khon Kaen University, Khon Kaen, 40002 Thailand; 6 Department of Mathematics, Abdul Wali Khan University Mardan, Khyber Pakhtunkhwa, 23200 Pakistan

**Keywords:** Energy science and technology, Mathematics and computing, Nanoscience and technology

## Abstract

Ethylene glycol is commonly used as a cooling agent in the engine, therefore the study associated with EG has great importance in engineering and mechanical fields. The hybrid nanofluid has been synthesized by adding copper and graphene nanoparticles into the Ethylene glycol, which obeys the power-law rheological model and exhibits shear rate-dependent viscosity. As a result of these features, the power-law model is utilized in conjunction with thermophysical characteristics and basic rules of heat transport in the fluid to simulate the physical situations under consideration. The Darcy Forchhemier hybrid nanofluid flow has been studied under the influence of heat source and magnetic field over a two-dimensionally stretchable moving permeable surface. The phenomena are characterized as a nonlinear system of PDEs. Using resemblance replacement, the modeled equations are simplified to a nondimensional set of ODEs. The Parametric Continuation Method has been used to simulate the resulting sets of nonlinear differential equations. Figures and tables depict the effects of physical constraints on energy, velocity and concentration profiles. It has been noted that the dispersion of copper and graphene nanoparticulate to the base fluid ethylene glycol significantly improves velocity and heat conduction rate over a stretching surface.

## Introduction

Industrial and natural fluids detract from Newton's viscosity law because their viscosity does not correlate with deformation rate. These fluids are recognized as non-Newtonian fluids (NNF). They are further subdivided into numerous classes. There are some notorious classes of NNF, whose shear rate is not independent of flow direction. Shear rate-dependent viscosity fluids are subdivided further Herschel–Bulkley, Casson fluids, Bingham fluids, Carreau-Yasuda fluid, Carreau fluids and power-law fluid^1–6^. Minakov et al.^7^ described the findings of a practical assessment of the nanofluids (NF) flow incorporating nano particulates of various metals and oxides. Al-Mubaddel et al.^8^ has utilized the rheological model to predict the energy and mass transfer via hybrid NF fluid subjected to a magnetic field. The findings revealed that the transport of species in a power-law fluid is influenced by the opposite trends of chemical reactions. Using a power law fluid model, Cheng^9^ studied the energy and mass transmission. A porous, parallel plate microreactor with NN working fluid was inspected by Javidi et al.^10^ with the transfer of heat and mass, as well as thermodynamic irreversibility. The results show that Microreactor thermodynamic irreversibility is shown to be affected by wall thickness and thermal variance. Chavaraddi et al.^11^ examined the thermal properties of a passive conducting fluid in a bounded domain subjected to external effects. The results exposed that a power-law fluid stabilize the system while an electric field or magnetic field destabilizes the interface. Alsallami et al.^12^ develop an Maxwell nanofluid flow with arrhenius activation energy over a rotating disk. The dynamics of a bubble confined within an elastic solid in a non-Newtonian power-law fluid are investigated by Arefmanesh et al.^13^. The numerical examination of 2D non-Newtonian power-law fluids through a circular cylinder is presented by Bilal et al.^14^. The conclusions show that the system's frequency and apparent viscosity have a significant impact on the VIV of the cylinder's properties. Elattar et al.^15^ studied the power-law fluid flows past over a porous slender stretching surface. Some recent contributions have been made by many researchers towards power-law fluid flow through porous enlarging surface^16–19^.

Heat and mass transfer are utilized in a wide range of industries, including heating and air conditioning, energy systems, cars, steam-electric power production, disease detection and electronic device cooling^20–24^. Ordinary fluid, on the other hand, is unable to meet this requirement of heat transition. As a result, the incorporation of NPs in the base fluid is very suggestive. Nanofluids have gotten a lot of attention in the previous decade, especially in heat transfer enhancement and renewable energy. Nanofluids are used in ocean power plants, thermodynamics, solar collectors, hydropower rotors, geothermal heat exchangers and wind turbines^25–30^. Jia et al.^31^ described a realistic approach for synthesizing cell membrane (CM)-coated iron oxide nanoclusters as a nanocarrier for anticancer medicine Iron oxide nanoparticles were used by Schwaminger et al.^32^ to design novel flotation process approaches that took use of their particular features. An alkaline coprecipitation procedure was used to create magnetic nanoparticles with a basic crystal structure of 9 nm, which were subsequently coated with sulfate. A study conducted by Bilal et al.^33^ considered the impacts of Hall current on the flow of carbon nanotubes and iron ferrite hybrid nanoliquids along the surface of a spinning disc.

The C–C bond makes CNTs in base fluid more effective than other nanocomposite forms. The desired outcome can be obtained by covalent or non-covalent manipulation of CNT nanofluid^34–36^. Ferrofluid flow across an endless, impermeable disc was analyzed by Tassaddiq et al.^37^. They found that the combination of CNTs and Fe_2_O_3_ nanoparticles significantly increases the transfer of energy and mass. CNT + Fe_2_O_3_/H_2_O has a stronger impact on carrier fluid thermophysical parameters than magnetic ferrite nanoparticles. Bilal et al.^38^ scrutinized the cumulative upshots of electro- and magneto-hydrodynamics on the flow of water-based hybrid nanofluids over two collateral sheets in a two-collateral sheet arrangement Ullah et al.^39^ numerically investigated an unstable squeezed flow of a HNF comprising CNTs and CuO, as well as a nanofluid containing CNTs. In an experimental context, Alharbi et al.^40^ calculated the upshot of particle and energy concentration on the dynamic of hybrid nanoliquid. The results show that by increasing the number of nanoparticles, the viscosity increases up to 168 percent. Ullah et al.^41^ investigated how a hybrid nanofluid flow on the outside of a revolving disc could improve mass and heat conduction. They observed that hybrid nanofluids are more successful at transporting heat than regular nanofluids^42–46^. discuss the further uses, synthesis, utilization, and structural characteristics of magnetic nanomaterials and carbon nanotubes.

It has been revealed that fluids with shear rate-dependent viscosity can return to their thermal equilibrium state as a result of the thermal relaxation time features of the fluids^47,48^. Heat and mass transmission through convective Maxwell nanoliquid across an extending sheet was studied by Sui et al.^49^ using the Cattaneo–Christov (CC) double-diffusion model. The results show that this viscoelastic relaxation framework system predicts relaxation timings transport properties. The CC theory is used by Hafeez et al.^50^ instead of traditional Fourier's and Fick's laws to investigate the energy propagation in the fluid. Manjunatha et al.^51^ and Naveen et al.^52^ inspected the energy and mass transportation mechanisms caused by a chemical reaction, thermophoresis effect and Brownian motion in a stream of viscous nanocomposites submitted to a curvy stretched surface. Madhukesh et al.^53^ considered CC double diffusion models to investigate 3D Prandtl liquid flow. Similar studies related to the proposed model can be found in refs^54,55^.

The literature revealed that no investigation on the argumentation of energy transmission in a power-law fluid due to the combined dispersion of Cu and Graphene nanoparticles has been found. The motive of the research is to develop a computational model using copper and Graphene nanoparticles in the carrier fluid ethylene glycol, to magnify the energy communication rate and boost the competence and ability of thermal energy transference for a variety of biological and commercial purposes. Furthermore, the PCM procedure has been used to tackle the modeled equations of the proposed model.

## Mathematical formulation

**Figure 1:**
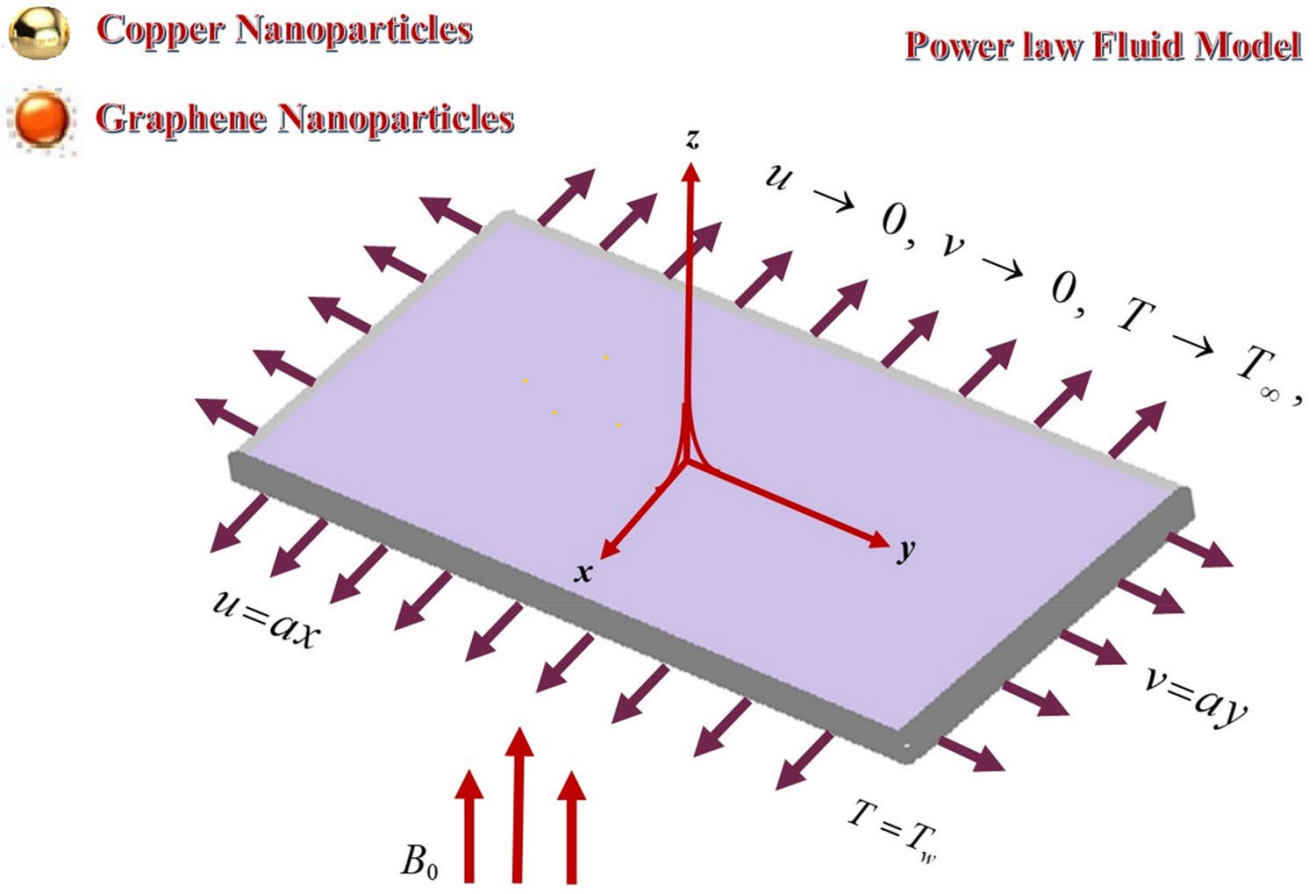
Hybrid nanofluid flow over a moving permeable surface.

We considered a 2D HNF flow over a stretchable moving surface with fixed temperature $T_{w}$ with velocity $V_{w} = ax\hat{i} + by\hat{j}$, the sheet surface is moving. The non- Newtonian hybrid nanofluid fluid, flow over the surface, obeys the power-law rheological model. Figure 1 revealed the physical mechanism of the fluid flow over a moving permeable surface. The energy transport mechanism is supposed to be augmented with the dispersion of nanoparticles Copper and Graphene. The wall of sheet is moving with 2D velocity, but as a result the heat transfer and of fluid flow will be 3D. The basic equation, which regulates the fluid flow and energy transport mechanism are as follow^27,56,57^:

Continuity equation

1 $$\frac{\partial u}{{\partial x}} + \frac{\partial v}{{\partial y}} + \frac{\partial w}{{\partial z}} = 0,$$

Momentum equation along *x*-axis

2 $$u\frac{\partial u}{{\partial x}} + v\frac{\partial u}{{\partial y}} + w\frac{\partial u}{{\partial z}} = \frac{{k_{1} }}{{\rho_{hnf} }}\frac{\partial }{\partial z}\left( {\left| {\frac{\partial u}{{\partial z}}} \right|^{n - 1} \frac{\partial u}{{\partial z}}} \right) - \left( {\frac{{\sigma_{hnf} B_{0}^{2} }}{{\rho_{hnf} }}(u - v)} \right) - \frac{{\upsilon_{hnf} }}{{K^{*} }}u - \frac{1}{{\rho_{hnf} }}Fu^{2} ,$$

Momentum equation along *y*-axis

3 $$u\frac{\partial v}{{\partial x}} + v\frac{\partial v}{{\partial y}} + w\frac{\partial v}{{\partial z}} = \frac{{k_{1} }}{{\rho_{hnf} }}\frac{\partial }{\partial z}\left( {\left| {\frac{\partial u}{{\partial z}}} \right|^{n - 1} \frac{\partial v}{{\partial z}}} \right) - \left( {\frac{{\sigma_{hnf} B_{0}^{2} }}{{\rho_{hnf} }}(u + v)} \right) - \frac{{\upsilon_{hnf} }}{{K^{*} }}v - \frac{1}{{\rho_{hnf} }}Fv^{2} ,$$

Energy equation

4 $$\begin{aligned} u\frac{\partial T}{{\partial x}} + v\frac{\partial T}{{\partial y}} + w\frac{\partial T}{{\partial z}} &+ \lambda_{1} \left( \begin{gathered} u^{2} \frac{{\partial^{2} T}}{{\partial x^{2} }} + w^{2} \frac{{\partial^{2} T}}{{\partial z^{2} }} + v^{2} \frac{{\partial^{2} T}}{{\partial y^{2} }} + 2vw\frac{{\partial^{2} T}}{\partial y\partial z} + 2uv\frac{{\partial^{2} T}}{\partial x\partial y} + 2uw\frac{{\partial^{2} T}}{\partial x\partial z} \hfill \\ + \left( {u\frac{\partial u}{{\partial x}} + w\frac{\partial u}{{\partial z}} + v\frac{\partial u}{{\partial y}}} \right)\frac{\partial T}{{\partial x}} - \frac{{Q_{0} }}{{(\rho C_{p} )_{hnf} }}\left( {u\frac{\partial T}{{\partial x}} + w\frac{\partial T}{{\partial z}} + v\frac{\partial T}{{\partial y}}} \right) \hfill \\ + \left( {u\frac{\partial v}{{\partial x}} + w\frac{\partial v}{{\partial z}} + v\frac{\partial v}{{\partial y}}} \right)\frac{\partial T}{{\partial y}} + \left( {u\frac{\partial w}{{\partial x}} + w\frac{\partial w}{{\partial z}} + v\frac{\partial w}{{\partial y}}} \right)\frac{\partial T}{{\partial z}} \hfill \\ \end{gathered} \right) \\ &\quad = \frac{{k_{hnf} }}{{(\rho C_{p} )_{hnf} }}\frac{{\partial^{2} T}}{{\partial y^{2} }} + \frac{{Q_{0} (T - T_{\infty } )}}{{(\rho C_{p} )_{hnf} }}. \\ \end{aligned}$$

5 $$\left. \begin{gathered} u = ax = U_{w} ,\,\,\,\,\,v = by = V_{w} ,\,\,\,\,T - T_{w} = 0,\,\,\,\,w\, = 0\,\,\,\,\,{\text{as}}\,\,\,\,z = 0,\, \hfill \\ u \to 0,\,\,\,\,\,\,v \to 0,\,\,\,\,T \to T_{\infty } \,\,\,\,\,{\text{as}}\,\,\,\,\,z \to \infty . \hfill \\ \end{gathered} \right\}$$

here $U_{w}$ and $V_{w}$ represents that the surface is stretching along both *x*-axis and *y*-axis directions.

## Similarity transformation

The similarity variables are defined as^59^:

6 $$\begin{gathered} \,u = axf^{\prime } (\eta ),\,\,\,w = - a\left( {\frac{{ba^{n - 2} }}{{\rho_{f} }}} \right)^{{\frac{1}{n + 1}}} \left( {\frac{2n}{{n + 1}}f(\eta ) + \frac{1 - n}{{1 + n}}\eta f^{\prime}(\eta ) + g(\eta )} \right)x^{{\frac{n - 1}{{n + 1}}}} ,\,\,\,v = ybg^{\prime } (\eta ),\,\, \hfill \\ \,\theta (\eta ) = \frac{{T - T_{\infty } }}{{T_{w} - T_{\infty } }},\,\,\eta = z\left( {\frac{{ba^{n - 2} }}{{\rho_{f} }}} \right)^{{\frac{1}{n + 1}}} x^{{\frac{1 - n}{{1 + n}}}} \hfill \\ \end{gathered}$$

Incorporating Eq. (6) into Eqs. (1–4), we get:

7 $$\begin{aligned} & \left( {\left| {f^{\prime \prime } } \right|^{n - 1} f^{\prime } } \right)^{\prime } - \left( {1 - \varphi_{2} } \right)\left( {\left( {1 - \varphi_{1} } \right) + \varphi_{1} \frac{{\rho_{s1} }}{{\rho_{f} }}} \right) + \varphi_{2} \frac{{\rho_{s2} }}{{\rho_{f} }}\left[ {Frf^{\prime 2} + \left( {\frac{2n}{{n + 1}}f + g} \right)f^{\prime \prime } } \right] \\ & \quad - \;(1 - \varphi_{1} )^{2.5} (1 - \varphi_{2} )^{2.5} \frac{{\sigma_{hnf} }}{{\sigma_{f} }}M^{2} (\lambda f^{\prime } - g^{\prime } ) = 0, \\ \end{aligned}$$

8 $$\begin{aligned} & \left( {\left| {f^{\prime \prime } } \right|^{n - 1} g^{\prime \prime } } \right)^{\prime } - (1 - \varphi_{2} )\left[ {(1 - \varphi_{1} ) + \varphi_{1} \frac{{\rho_{s1} }}{{\rho_{f} }}} \right] + \varphi_{2} \frac{{\rho_{s2} }}{{\rho_{f} }}\left[ {Frg^{\prime 2} + \left( {\frac{2n}{{n + 1}}f + g} \right)g^{\prime \prime } } \right] \\ & - (1 - \varphi_{1} )^{2.5} (1 - \varphi_{2} )^{2.5} \frac{{\sigma_{hnf} }}{{\sigma_{f} }}M^{2} (2\lambda g^{\prime } ) = 0, \\ \end{aligned}$$

9 $$\begin{aligned} & \theta^{\prime \prime } + \frac{{k_{f} (\rho C_{p} )_{hnf} }}{{k_{hnf} (\rho C_{p} )_{f} }}\left[ {Pr\left( {\frac{2n}{{n + 1}}} \right)f\theta^{\prime } + Prg\theta } \right] + \frac{{k_{f} }}{{k_{hnf} }}h_{s} \left( {\frac{2n}{{n + 1}}} \right)Pr\theta \\ & \quad - \;\frac{{k_{f} (\rho C_{p} )_{hnf} }}{{k_{hnf} (\rho C_{p} )_{f} }}Pr\lambda_{E} \left\{ {\left( {\frac{2n}{{n + 1}}f + g} \right)\left( {\frac{2n}{{n + 1}}f^{\prime } + g^{\prime } } \right)\theta^{\prime } \left( {\frac{2n}{{n + 1}}f + g} \right)^{2} \theta^{\prime \prime } + h_{s} Pr\left( {\frac{2n}{{n + 1}}f + g} \right)\theta^{\prime } } \right\} = 0. \\ \end{aligned}$$

Incorporating $\wp_{1} ,\,\,\,\wp_{2} ,\,\,\,\wp_{3}$ in Eqs. (7–9) for simplification purpose, where $\wp_{1} ,\,\,\,\wp_{2} ,\,\,\,\wp_{3}$ are defined as:

$$\wp_{1} = \left( {1 - \varphi_{2} } \right)\left( {\left( {1 - \varphi_{1} } \right) + \varphi_{1} \frac{{\rho_{s1} }}{{\rho_{f} }}} \right) + \varphi_{2} \frac{{\rho_{s2} }}{{\rho_{f} }},\,\,\,\wp_{2} = \left( {1 - \varphi_{1} } \right)^{2.5} \left( {1 - \varphi_{2} } \right)^{2.5} \frac{{\sigma_{hnf} }}{{\sigma_{f} }},\,\,\,\wp_{3} = \frac{{k_{f} (\rho C_{p} )_{hnf} }}{{k_{hnf} (\rho C_{p} )_{f} }}.$$

we get

10 $$\left( {\left| {f^{\prime \prime } } \right|^{n - 1} f^{\prime \prime } } \right)^{\prime } - \wp_{1} \left( {Frf^{\prime 2} + \left( {\frac{2n}{{n + 1}}f + g} \right)f^{\prime \prime } } \right) - \wp_{2} M^{2} \left( {\lambda f^{\prime } - g^{\prime } } \right) = 0,$$

11 $$\left( {\left| {f^{\prime \prime } } \right|^{n - 1} g^{\prime \prime } } \right)^{\prime } - \wp_{1} \left( {Frg^{\prime 2} + \left( {\frac{2n}{{n + 1}}f + g} \right)g^{\prime \prime } } \right) - \wp_{2} M^{2} \left( {2\lambda g^{\prime } } \right) = 0,$$

12 $$\left. \begin{aligned} & \theta^{\prime \prime } + \wp_{3} \left( {Pr\left( {\frac{2n}{{n + 1}}} \right)f\theta^{\prime \prime } + Pr\theta g} \right) + \frac{{k_{f} }}{{k_{hnf} }}h_{s} \left( {\frac{2n}{{n + 1}}} \right)Pr\theta - \wp_{3} Pr\lambda_{E} \\ & \left\{ {\left( {\frac{2n}{{n + 1}}f + g} \right)\left( {\frac{2n}{{n + 1}}f^{\prime } + g^{\prime } } \right)\theta^{\prime}\left( {\frac{2n}{{n + 1}}f + g} \right)^{2} \theta^{\prime \prime } + h_{s} Pr\left( {\frac{2n}{{n + 1}}f + g} \right)\theta^{\prime } } \right\} = 0. \\ \end{aligned} \right\}$$

The boundary conditions are given below:

13 $$\left. \begin{gathered} f(0) = 0,\,\,\,f^{\prime } (0) = \,g^{\prime } (0) = 1,\,\,g(0) = 0,\,\,\,\,\theta (0) = 1 \hfill \\ f(\infty ) = 0,\,\,\,\,g(\infty ) = 0,\,\,\,\,\,\theta (\infty ) = 0. \hfill \\ \end{gathered} \right\}$$

here $M^{2} = \frac{{2\sigma_{f} B_{0}^{2} }}{{a\rho_{f} }}$ is the Hartmann number, $h_{s} = \frac{Q}{{a(C_{p} )_{f} \rho_{f} }}$ is the heat generation term, $\lambda_{E} = \frac{{a\lambda_{1} }}{x}$ is the thermal relaxation constraint, $Re = \frac{{x^{n} (U_{w} )^{2 - n} \rho_{f} }}{{k_{f} }}$ is the Reynolds number, $Pr = \frac{{(C_{p} )\rho_{f} ax^{2} {\text{Re}}^{{\frac{2}{n + 1}}} }}{{k_{f} }}$ is the Prandtl number and $Fr = \frac{{xC_{b} }}{{\sqrt {K^{*} } }}$ is the Darcy Forchhemier number. where, $s_{1}$ and $s_{2}$ represent the Copper and Graphene nanoparticles respectively.

The skin friction expressed as:

14 $$\frac{1}{2}C_{f} (Re)^{{\frac{1}{n + 1}}} = \frac{{\left| {f^{\prime \prime } (0)} \right|^{n} }}{{(1 - \varphi_{1} )^{2.5} (1 - \varphi_{2} )^{2.5} }},\,\,\frac{1}{2}C_{g} (Re)^{{\frac{1}{n + 1}}} = \frac{{\left| {f^{\prime \prime } (0)} \right|^{n - 1} g^{\prime \prime } (0)}}{{(1 - \varphi_{1} )^{2.5} (1 - \varphi_{2} )^{2.5} }}.$$

The heat transfer rate can be stated as:

15 $$Nu = \frac{{xq_{w} }}{{K_{f} (T_{w} - T_{\infty } )}},\,\,\,q_{w} = \left. { - k_{hnf} \frac{\partial T}{{\partial z}}} \right|_{{{\text{at}}\,\,{\text{wall}}}} ,\,\,\,({\text{Re}} )^{0.5} Nu = \frac{{k_{hnf} }}{{k_{f} }}\theta^{\prime}(0).$$

## Numerical solution

The main steps, while dealing with PCM method are^60–63^:

*Step 1* Converting the system of BVP to the ODEs

16 $$\left. \begin{gathered} \hbar_{1} (\eta ) = f(\eta ),\,\,\hbar_{2} (\eta ) = f^{\prime } (\eta ),\,\,\,\hbar_{3} (\eta ) = g(\eta ),\,\, \hfill \\ \hbar_{4} (\eta ) = g^{\prime } (\eta ),\,\,\hbar_{5} (\eta ) = \theta (\eta ),\,\,\hbar_{6} (\eta ) = \theta^{\prime } (\eta ).\, \hfill \\ \end{gathered} \right\}$$

By putting Eq. (16) in Eq. (10–13), we get:

17 $$\left( {\left| {\hbar^{\prime }_{2} } \right|^{n - 1} \hbar^{\prime }_{2} } \right)^{\prime } - \wp_{1} \left( {Fr\hbar_{2}^{2} + \left( {\frac{2n}{{n + 1}}\hbar_{1} + \hbar_{3} } \right)\hbar^{\prime }_{2} } \right) - \wp_{2} M^{2} \left( {\lambda \hbar_{2} - \hbar_{4} } \right) = 0,$$

18 $$\left( {\left| {\hbar^{\prime}_{2} } \right|^{n - 1} \hbar^{\prime}_{4} } \right)^{\prime } - \left( {\wp_{1} Fr\hbar_{4}^{{}} + 2\lambda \wp_{2} M^{2} } \right)\hbar_{4} - \wp_{1} \left( {\frac{2n}{{n + 1}}\hbar_{1} + \hbar_{3} } \right)\hbar^{\prime}_{4} = 0,$$

19 $$\begin{aligned} & \hbar^{\prime }_{6} - \wp_{3} Pr\lambda_{E} \left\{ \begin{gathered} \wp_{3} Pr\left( {\frac{2n}{{n + 1}}} \right)\hbar_{1} + \left( {\hbar_{1} \frac{2n}{{n + 1}} + \hbar_{3} } \right)\left( {\frac{2n}{{n + 1}}\hbar_{2} + \hbar_{4} } \right) \hfill \\ \left( {\frac{2n}{{n + 1}}\hbar_{1} + \hbar_{3} } \right)^{2} \hbar^{\prime }_{6} + h_{s} Pr\left( {\frac{2n}{{n + 1}}\hbar_{1} + \hbar_{3} } \right) \hfill \\ \end{gathered} \right\}\hbar_{6} \\ & + \wp_{3} Pr\hbar_{5} \hbar_{3} + \frac{{k_{f} }}{{k_{hnf} }}h_{s} \left( {\frac{2n}{{n + 1}}} \right)Pr\hbar_{5} = 0. \\ \end{aligned}$$

the boundary conditions are:

20 $$\left. \begin{gathered} \hbar_{1} (0) = \hbar_{3} (0) = 0,\,\,\,\hbar_{2} (0) = \,\hbar_{4} (0) = 1,\,\,\hbar_{5} (0) = 1 \hfill \\ \hbar_{1} (\infty ) = \hbar_{3} (\infty ) = 0,\,\,\,\,\,\hbar_{5} (\infty ) = 0. \hfill \\ \end{gathered} \right\}$$

*Step 2* Introducing the embedding parameter p in Eqs. (17–19):

21 $$\left( {\left| {\hbar^{\prime }_{2} } \right|^{n - 1} \hbar^{\prime }_{2} } \right)^{\prime } - \left( {\wp_{1} Fr\hbar_{2}^{{}} + \wp_{2} M^{2} \lambda } \right)(\hbar_{2} - 1)p + \wp_{2} M^{2} \hbar_{4} - \wp_{1} \left( {\hbar_{1} \frac{2n}{{n + 1}} + \hbar_{3} } \right)\hbar^{\prime }_{2} = 0,$$

22 $$\left( {\left| {\hbar^{\prime }_{2} } \right|^{n - 1} \hbar^{\prime }_{4} } \right)^{\prime } - \left( {\wp_{1} Fr\hbar_{4}^{{}} + 2\lambda \wp_{2} M^{2} } \right)(\hbar_{4} - 1)p - \wp_{1} \left( {\frac{2n}{{n + 1}}\hbar_{1} + \hbar_{3} } \right)\hbar^{\prime }_{4} = 0,$$

23 $$\begin{gathered} \hbar^{\prime}_{6} - \wp_{3} Pr\lambda_{E} \left\{ \begin{gathered} \wp_{3} Pr\left( {\frac{2n}{{n + 1}}} \right)\hbar_{1} + \left( {\frac{2n}{{n + 1}}\hbar_{1} + \hbar_{3} } \right)\left( {\frac{2n}{{n + 1}}\hbar_{2} + \hbar_{4} } \right) \hfill \\ \left( {\frac{2n}{{n + 1}}\hbar_{1} + \hbar_{3} } \right)^{2} \hbar^{\prime}_{6} + h_{s} Pr\left( {\frac{2n}{{n + 1}}\hbar_{1} + \hbar_{3} } \right) \hfill \\ \end{gathered} \right\}(\hbar_{6} - 1)p + \hfill \\ \wp_{3} Pr\hbar_{5} \hbar_{3} + \frac{{k_{f} }}{{k_{hnf} }}h_{s} \left( {\frac{2n}{{n + 1}}} \right)Pr\hbar_{5} = 0. \hfill \\ \end{gathered}$$

*Step 3* Differentiating by parameter ’p’

24 $$V^{\prime } = \rlap{--} \Delta V + R,$$

where $\rlap{--} \Delta$ is the coefficient matrix.

25 $$V = \frac{{d\hbar_{i} }}{d\tau }$$

where *i* = 1*,* 2*,*…11*.*

*Step 4* Apply the Cauchy Principal

26 $$V = aU + W,$$

where W and U are the indefinite vector functions.

27 $$U^{\prime } = aU,$$

28 $$W^{\prime } = \rlap{--} \Delta W + R,$$

Using Eq. (26) in Eq. (24), we get

29 $$(aU + W)^{\prime } = \rlap{--} \Delta (aU + W) + R,$$

*Step 5* Solving the Cauchy problems

30 $$\frac{{U^{i + 1} - U^{i} }}{\Delta \eta } = \rlap{--} \Delta U^{i + 1} ,\,\,\,\,\frac{{W^{i + 1} - W^{i} }}{\Delta \eta } = \rlap{--} \Delta W^{i + 1} .$$

Finally, we get:

31 $$U^{i + 1} = (I - \Delta \rlap{--} \Delta \eta )^{ - 1} U^{i} ,\,\,\,\,\,\,\,\,W^{i + 1} = (I - \Delta \rlap{--} \Delta \eta )^{ - 1} (W^{i} + \Delta \eta R).$$

## Results and discussion

This section explains the physical mechanism behind each result, which is shown in the Figures and Table.

**Figure 2:**
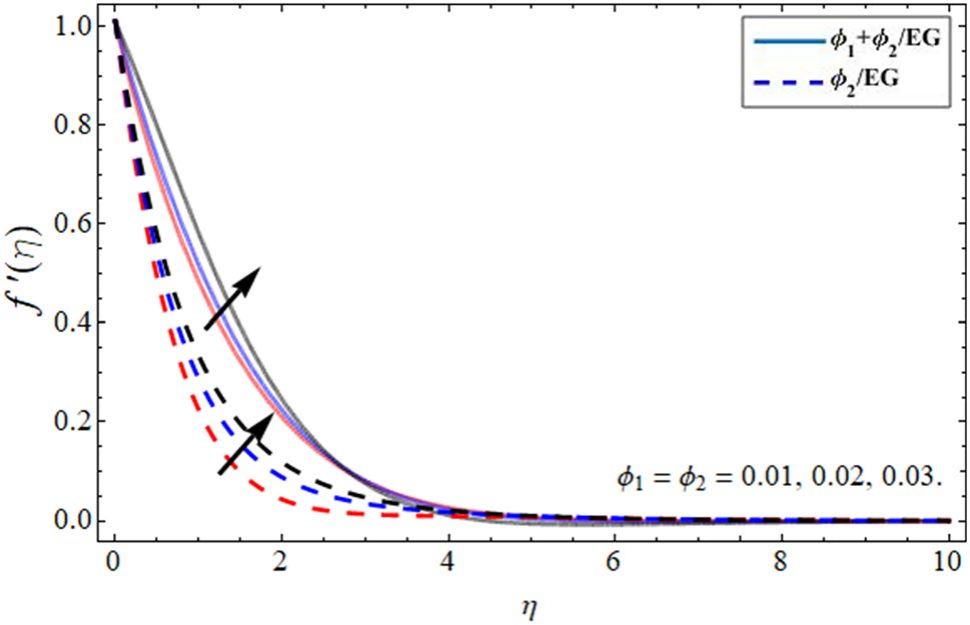
Velocity $f^{\prime } \left( \eta \right)$ outlines versus the effect of nanoparticles volume friction $\phi$.

**Figure 3:**
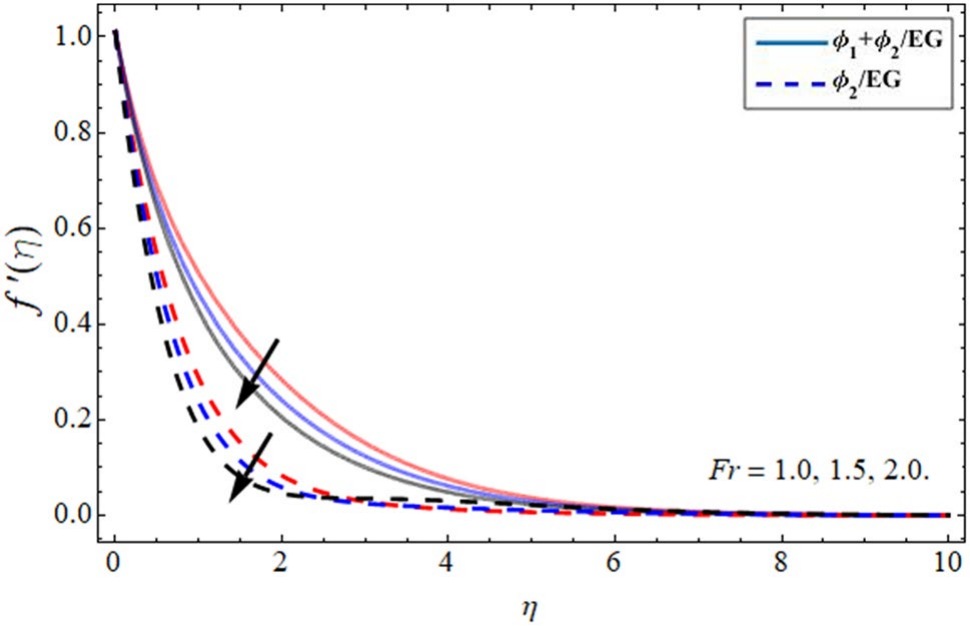
Velocity $f^{\prime } \left( \eta \right)$ outlines versus the effect of Darcy Forchhemier term *Fr***.**

**Figure 4:**
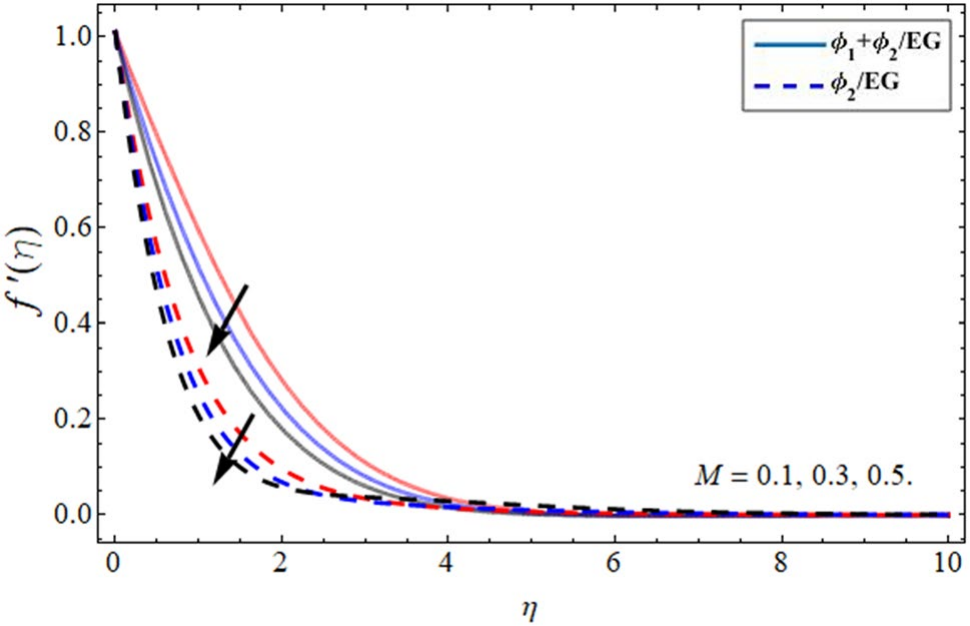
Velocity $f^{\prime}\left( \eta \right)$ outlines versus the effect of magnetic term *M***.**

**Figure 5:**
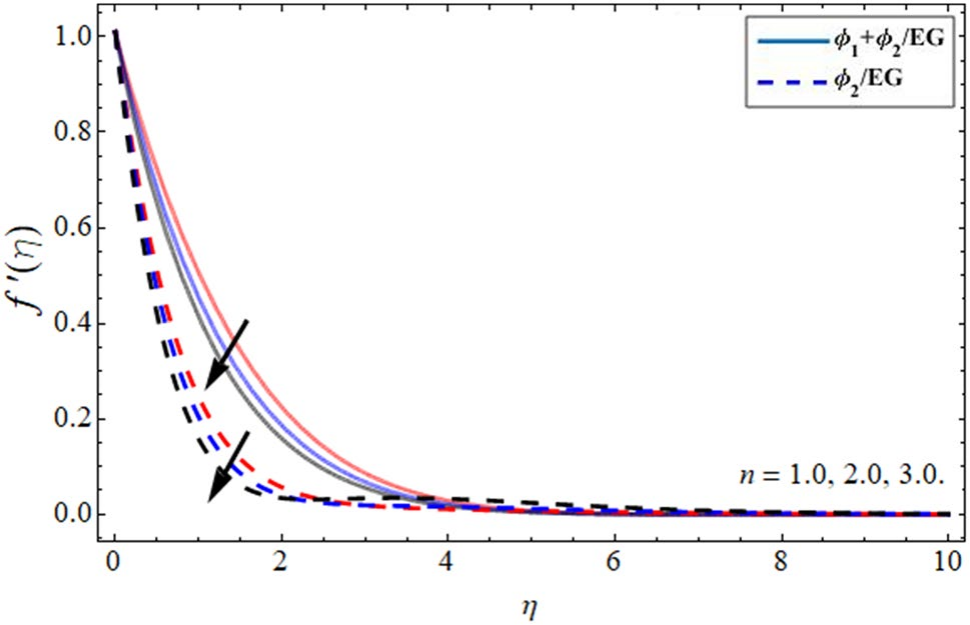
Velocity $f^{\prime } \left( \eta \right)$ outlines versus the effect of parameter *n***.**

Figures 2, 3, 4 and 5 revealed the conduct of velocity contour $f^{\prime } \left( \eta \right)$ versus the variation of nanoparticles volume friction $\phi$, Darcy Forchhemier term *Fr*, magnetic term *M* and parameter *n* (for $n = 1,$ the fluid behave as Newtonian, while at $n > 1,$ the shear thicking phenomena occur) respectively. Figure 2 reported that the axial velocity outline boosts with the addition of hybrid NPs in EG. Physically, the specific heat capacity of ethylene glycol is greater than copper and graphene NPs, so the addition of nanoparticles declines its thermal absorbing capability, as a result, the fluid velocity enhances. Figure 3 signifies that the axial velocity $f^{\prime } \left( \eta \right)$ reduces with the flourishing Darcy effect. Figure 4 manifested that the fluid velocity lessens with the rising effect of the magnetic field, because the resistive force, which is generated due to magnetic force contests the fluid flow. The fluid velocity declines under the consequences of parameter *n* as shown in Fig. 5.

**Figure 6:**
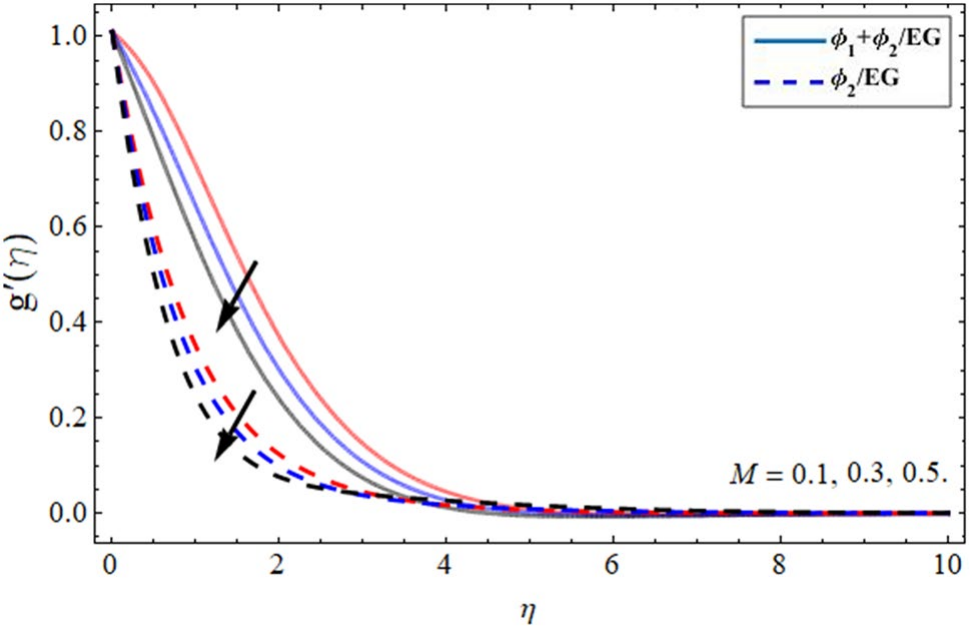
Velocity $g^{\prime } \left( \eta \right)$ outlines versus the effect of magnetic term *M***.**

**Figure 7:**
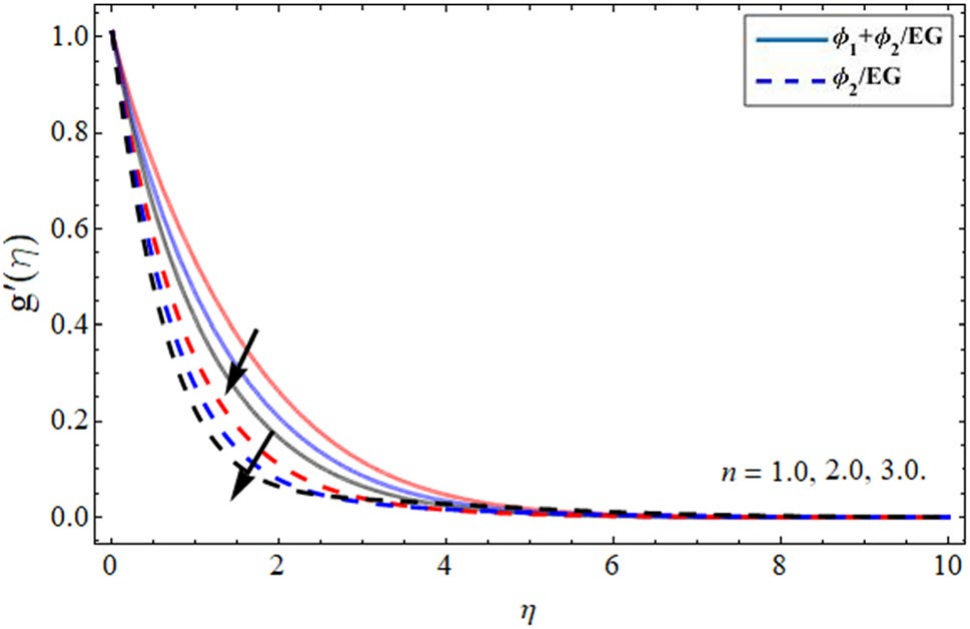
Velocity $g^{\prime } \left( \eta \right)$ outlines versus the effect of parameter *n***.**

Figures 6 and 7 illustrated that the velocity field in radial direction $g^{\prime } \left( \eta \right)$ diminishes with the growing values of magnetic effect and parameter n respectively. The Lorentz force formed due to magnetic term variation opposes the flow field, which costs in the lessening of the radial velocity field. Similarly, the increment of parameter *n* also reduces the momentum boundary layer as presented in Fig. 7.

**Figure 8:**
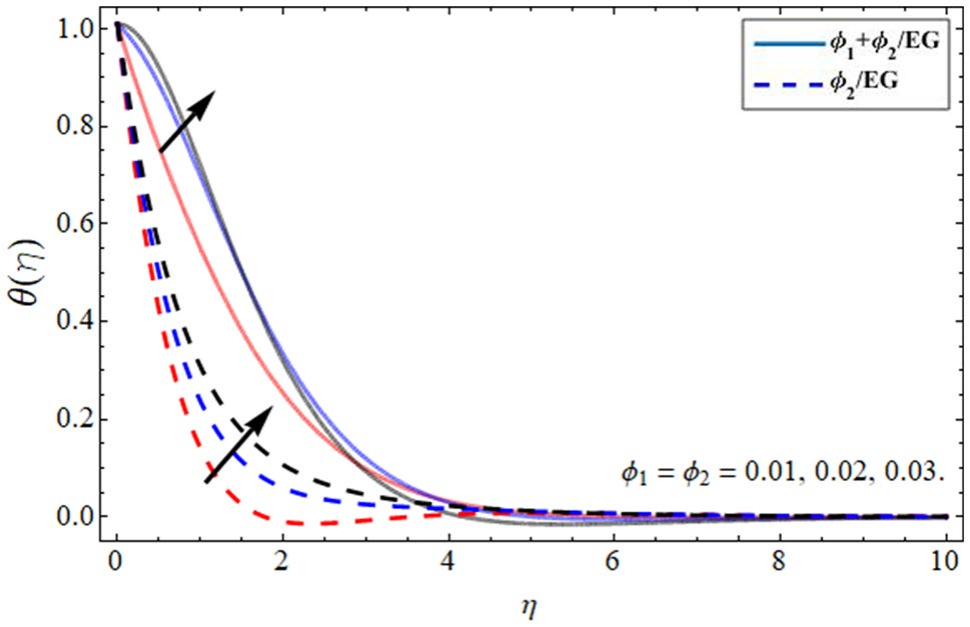
Energy $\theta \left( \eta \right)$ outlines versus the effect of nanoparticle volume friction $\phi$**.**

**Figure 9:**
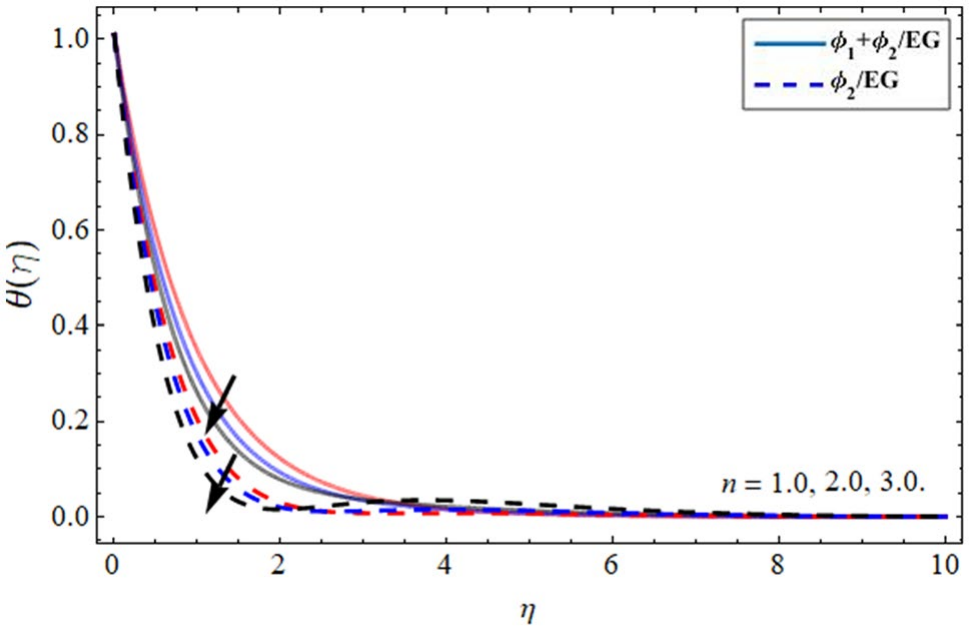
Energy $\theta \left( \eta \right)$ outlines versus the effect of parameter *n***.**

**Figure 10:**
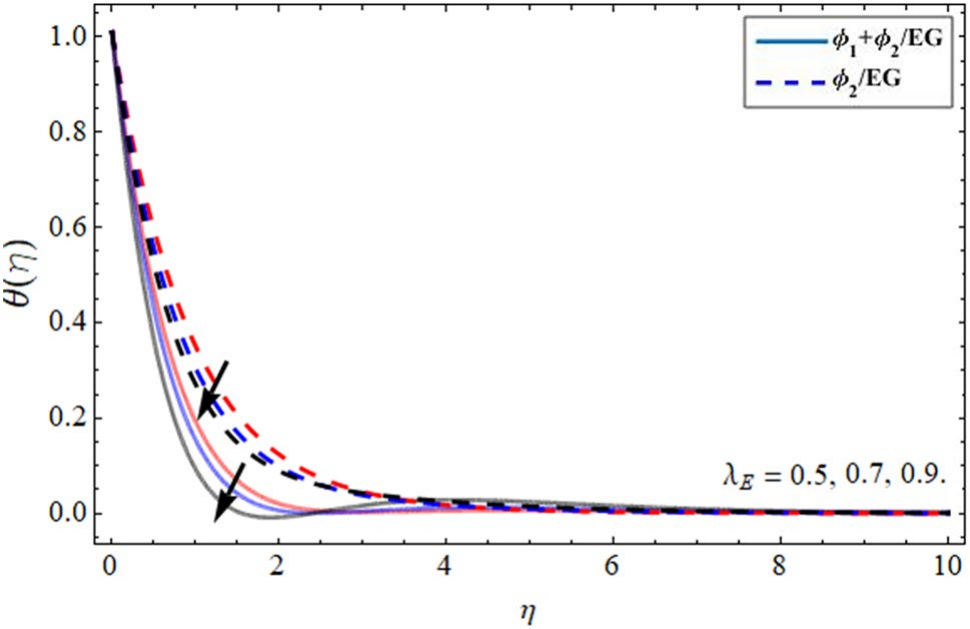
Energy $\theta \left( \eta \right)$ outlines versus the effect of thermal Deborah number $\lambda_{E}$**.**

**Figure 11:**
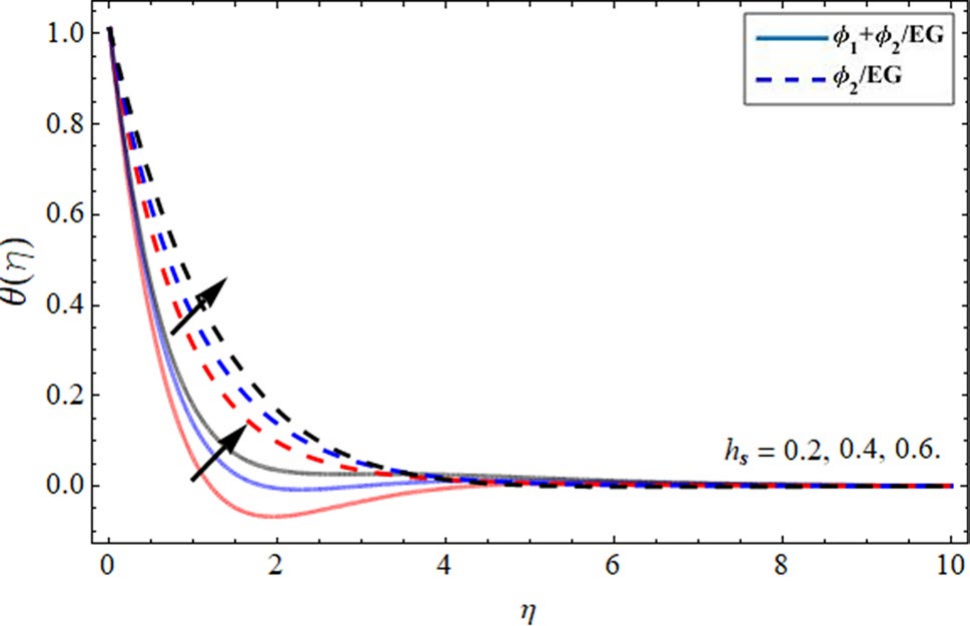
Energy $\theta \left( \eta \right)$ outlines versus the effect of heat generation term $h_{s}$**.**

Figures 8, 9, 10 and 11 revealed the presentation of energy contour against the flourishing trend of nanoparticulate volume friction $\phi$, parameter *n*, versus thermal Deborah number $\lambda_{E}$ and heat generation term $h_{s}$ respectively. Figure 8 elaborated that the variation of nanomaterials in the base fluid significantly magnifies its thermal conduction, which is more effective for industrial uses. The thermal conductivity of nanoparticles is greater than the ethylene glycol, that’s why its addition boosts the thermal property of the carrier fluid. Figure 9 demonstrated that the energy profile declines with the upshot of parameter *n*. Figure 10 and 11 display that the thermal energy outline decreases with the upshot of thermal Deborah number $\lambda_{E}$ while enhancing with the variation of heat source term *hs*. The heat source term augments the internal energy of the fluid, which encourages the fluid energy profile to enhance.

Table 1 reported the experimental values of nanoparticulate $\left( {\phi_{1} = \phi_{C} ,\,\,\phi_{2} = \phi_{Cu} } \right)$ and base fluid (ethylene glycol). Table 2 show the comparative analysis between nanofluid and hybrid nanofluid for skin friction along *x* $\left( { - ({\text{Re}} )^{{ - \frac{1}{2}}} C_{f} } \right)$ and *y* $\left( { - ({\text{Re}} )^{{ - \frac{1}{2}}} C_{g} } \right)$ direction and Nusselt number $\left( { - ({\text{Re}} )^{{ - \frac{1}{2}}} C_{Nu} } \right).$ It revealed that the energy conduction of hybrid nanofluid as compared to nanofluid is greater. The heat source and thermal Deborah number enhance the skin friction and Nusselt number remarkably. Table 3 shows the comparative evaluation of the published results with the current outcomes for skin friction and Nusselt number. It can be seen that the present results show closed adjustment with the existing literature, which ensure the validity of the results and proposed technique.

**Table 1:** The experimental values of base fluid (ethylene glycol) and nano particulates (copper and graphene^58^.

	$\rho \;(kg/m^{3} )$	$C_{p} \;(j/kgK)$	$k\;(W/mK)$	$\sigma \;(S/m)$
Ethylene glycol	1114	2415	0.252	5.5 × 10^−6^
Copper $\left( {\phi_{2} = \phi_{Cu} } \right)$	8933	385	401	5.96 × 10^7^
Graphene $\phi_{1} = \phi_{C}$	2250	2100	2500	1.0 × 107

**Table 2:** Statistical outcomes for physical quantities, such as skin friction $\left( { - (Re)^{{ - \frac{1}{2}}} C_{f} ,\,\, - (Re)^{{ - \frac{1}{2}}} C_{g} } \right)$ and Nusselt number $\left( { - (Re)^{{ - \frac{1}{2}}} C_{Nu} } \right)$ in case of both nanofluid and hybrid nanofluid.

	Nanofluid			Hybrid Nanofluid		
	$- (Re)^{{ - \frac{1}{2}}} C_{f}$	$- (Re)^{{ - \frac{1}{2}}} C_{g}$	$- (Re)^{{ - \frac{1}{2}}} C_{Nu}$	$- (Re)^{{ - \frac{1}{2}}} C_{f}$	$- (Re)^{{ - \frac{1}{2}}} C_{g}$	$- (Re)^{{ - \frac{1}{2}}} C_{Nu}$
***N***						
1.0	0.053003848	0.227327334	0.077382179	1.190488671	1.694350204	2.30917111
2.0	0.199883781	0.371279545	0.778005638	1.269149144	1.695060775	2.30830922
3,0	0.245493938	0.388009192	1.097857874	1.368321032	1.713010775	2.64498558
***M***						
0.0	0.344535715	0.447423914	1.334285223	0.292111797	0.358230717	1.42199998
0.2	0.427622087	0.516102601	1.267066226	0.178750768	0.138358081	1.33935047
0.4	0.606583762	0.680803897	1.101385769	0.062545122	0.015291717	1.25861093
$$\varvec{\lambda }_{\varvec{E}}$$						
0.0	0.727414454	0.686095548	0.389867706	0.416526708	0.679956092	5.07556644
0.6	0.857743852	0.719203214	1.353232227	0.526997918	0.731764147	5.09927908
1.1	0.886982272	0.751114887	1.314949813	0.580192798	0.802449387	4.17826838
$$\varvec{h}_{\varvec{s}}$$						
0.0	0.327677637	0.372891414	2.026693234	0.269941338	0.467279475	7.06024758
0.3	0.327677637	0.372891414	1.165464671	0.269941338	0.467279475	6.01929661
0.7	0.427677636	0.472891413	2.189427429	0.369941337	0.567279474	6.16243263

**Table 3:** Comparative assessment of published results with the current outcomes.

Parameters	$- (Re)^{{ - \frac{1}{2}}} C_{f}$	$- (Re)^{{ - \frac{1}{2}}} C_{f}$	$- (Re)^{{ - \frac{1}{2}}} C_{Nu}$	$- (Re)^{{ - \frac{1}{2}}} C_{Nu}$
***M***	Sadiq^59^	Present results	Sadiq^59^	Present results
0.0	0.4445357154	0.4445357455	2.434285223	2.434285423
0.3	0.5276220806	0.5276220932	2.367066226	2.367066441
0.7	0.7065837651	0.7065837753	2.201385769	2.201385873
***n***				
1.0	0.1530038477	0.153003868	0.0773821732	0.0773821753
2.0	0.2998837840	0.2998837952	1.878005638	1.878005852
3.0	0.3454939329	0.3454939651	2.197857874	2.197857976

## Conclusion

The three-dimensional Darcy Forchhemier hybrid nanofluid flow has been studied under the impact of heat generation and magnetic field over a two-dimensionally stretchable moving permeable surface. The phenomena are characterized as a nonlinear system of PDEs. The solution has been obtained through the PCM procedure. The key findings are:

- The accumulation of copper and graphene nanoparticulate to the base fluid ethylene glycol significantly improves velocity and heat conduction rate over a stretching surface.
- The axial velocity contour boosts with the addition of hybrid NPs in EG while reducing with the flourishing Darcy and magnetic effect.
- The variation of magnetic effect and parameter *n* diminishes the velocity field towards the radial direction $g^{\prime } \left( \eta \right)$.
- The thermal energy profile decreases with the effect of thermal Deborah number and parameter *n*. while enhancing with the variation of heat source term.
- As compared to the nanofluid (copper or graphene), hybrid nanofluid (copper + graphene) has a greater tendency for thermal energy conduction.
- The rising influence of heat source enhances the skin friction, while declines the Nusselt number.
- The effect of magnetic force also boosts the skin friction, while declines the Nusselt number.

## Data Availability

The data that supports the findings of this study are available within the article.
